# Proteomic profiles of the retina in an experimental unilateral optic nerve transection: Roles of Müller cell activation

**DOI:** 10.1002/ctm2.631

**Published:** 2022-04-26

**Authors:** Fancheng Yan, Xiaolei Wang, Xian Jiang, Yijie Chai, Jingxue Zhang, Qian Liu, Shen Wu, Yanling Wang, Ningli Wang, Shuning Li

**Affiliations:** ^1^ Department of Ophthalmology Beijing Friendship Hospital Capital Medical University Beijing China; ^2^ Department of Ophthalmology Anhui No. 2 Provincial People’s Hospital Hefei, Anhui Province China; ^3^ Department of Pathology School of Basic Medical Sciences Peking University Health Science Center Beijing China; ^4^ Beijing Institute of Ophthalmology Beijing Tongren Eye Center Beijing Tongren Hospital Capital Medical University Beijing Ophthalmology & Visual Sciences Key Laboratory Beijing China; ^5^ Beijing Institute of Brain Disorders Collaborative Innovation Center for Brain Disorders Capital Medical University Beijing China; ^6^ Department of Ophthalmology, Beijing Tongren Eye Center Beijing Tongren Hospital Capital Medical University Beijing Ophthalmology & Visual Sciences Key Laboratory Beijing China

Dear Editor:

Retinal ganglion cell (RGC) degeneration is a common pathogenesis in multiple ocular disorders and was studied in animal reproducible models of optic nerve transection (ONT) for the neuronal apoptosis in the adult central nervous system.^1^, ^2^, ^3^, ^4^, ^5^, ^6^ This directly affects RGCs as the main part of neuronal population apoptosis, although the exact molecular mechanisms remain unclear. The aim of our study is to explore proteomic profiles of retina in unilateral optic nerve transection and validate molecular interactions as new mechanisms. The “secondary degeneration” that RGC degeneration can be divided into two phases was hypothesized in acute and chronic eye diseases.^7^ The RGC axon damage resulted in intrinsic apoptosis triggered by activated retinal glial cells.^8^, ^9^ Müller cells are the most abundant glial cells in the retina and function like astrocytes in the brain. The current study furthermore evaluates changes in protein expression in the retina and the function of Müller glial cells during RGC degeneration, and assesses potential effects on RGC damage. Our data provide new evidence for understanding secondary optic nerve injury and new targets for precise therapy.

We established and validated the rat ONT model, quantified the retinal protein profiles using iTRAQ, and compared the protein expression between ONT and Controls 1, 4, 7, 14, and 28 days after ONT inducion. About 4717 proteins were detected, of which 54 were deferentially expressed proteins (DEPs) at five postoperative time points, including up‐regulated 25 (＞1.5‐fold) and down‐regulated 29 (＜0.67‐fold). About 708 DEPs were indentified within one postoperative time point (Figure S1). Figure 1A shows the top 10 up‐ and down‐regulated proteins at each time point. Of those proteins, the expression of Alb, Mgarp and Scrn2 was significantly up‐regulated, while Col1a1, Col14a1 and Dcn down‐regulated (Table 1). DEPs were further classified into biological process, cellular component and molecular function proteins using bioinformatics analysis (Figure 1B). The 708 DEPs were classified into 236 pathways according to the KEGG pathway database. The most abundant pathways included metabolic pathway, ribosome, carbon metabolism, Huntington's disease and spliceosome (Figure 1C). The protein–protein interactions of the 708 DEPs were grouped into six different clusters using the *K*‐means method (Figure 1D). The critical nodules within interaction networks of DEPs contains Cdc5l, C3, Ppp2rla, and Optn. The top 10 up‐ and down‐regulated DEPs was detected by hierarchical clustering at a time point/group after ONT induction (Figure 1E).

**FIGURE 1:**
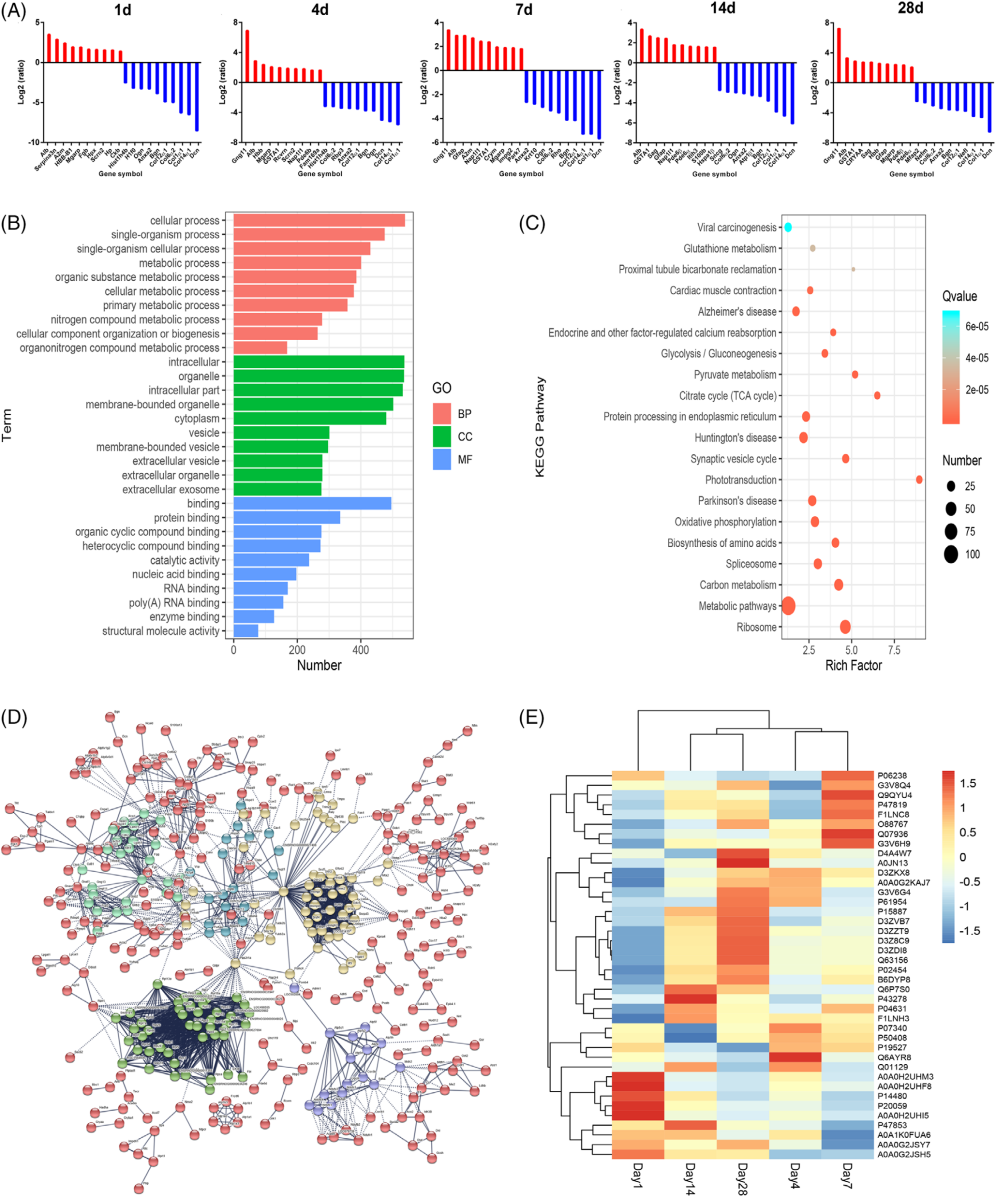
Proteomics and bioinformatics prediction. (A) The top 10 up‐regulated and down‐regulated differentially expressed proteins and fold changes in the retina of rats after optic nerve transection 1, 4, 7, 14, and 28 days after surgery. (B) Gene ontology (GO) analysis of 708 differentially expressed proteins detected in the study were categorised into the biological process (BP), cellular component (CC), and molecular function (MF). (C) The top 20 enriched KEGG pathways of differentially expressed proteins showed with bubble chart. (D) The protein‐protein interaction of 708 differentially expressed proteins analyzed by STRING. The network was classified into 6 clusters by *K*‐means method. The nodes represent protein in the network. (E) Hierarchical clustering analysis of the top 10 up‐regulated and down‐regulated differentially expressed proteins in the retina of rats after optic nerve transection at time points

**TABLE 1:** 54 differentially expressed proteins (DEPs) were consecutively altered in the retina of rats after ONT at the five time points

			ONT/control (fold change)				
Accession	Protein name	Gene symbol	1 d after ONT	4 d after ONT	7 d after ONT	14 d after ONT	28 d after ONT
Down‐regulation							
Q63156	Decorin (Fragment)	Q63156	.00223	.01486	.01536	.02077	.03293
P02454	Collagen alpha‐1(I) chain	Col1a1	.01369	.02859	.03236	.03795	.04426
D3ZZT9	Collagen type XIV alpha 1 chain	Col14a1	.01469	.02624	.02420	.03084	.03978
Q01129	Decorin	Dcn	.02134	.04279	.01666	.04150	.01205
F1LNH3	Collagen type VI alpha 2 chain	Col6a2	.02848	.10143	.09620	.12839	.09154
A0A0G2KAJ7	Collagen alpha‐1(XII) chain	Col12a1	.05718	.07436	.06818	.06733	.07399
Q07936	Annexin A2	Anxa2	.07961	.11598	.16246	.10020	.09331
D3ZVB7	Osteoglycin	Ogn	.09268	.09347	.11091	.13388	.15477
P47853	Biglycan	Bgn	.09383	.07255	.05176	.11786	.08047
G3V8H7	Olfactomedin‐like 3	Olfml3	.18007	.18060	.19388	.19408	.25997
D3Z952	Microfibril‐associated protein 2	Mfap2	.18127	.38142	.19513	.27472	.19324
A0A096P6L8	Fibronectin	Fn1	.18660	.25948	.22126	.21145	.24727
B3Y9H3	S100 calcium binding protein A10	S100A10	.22431	.26543	.29485	.24717	.25176
G3V8L3	Lamin A	Lmna	.26497	.31943	.35378	.32968	.38461
Q6P3E1	Rps16 protein (Fragment)	RPS16	.27947	.37640	.50116	.58969	.44949
C0JPT7	Filamin A	Flna	.32953	.25454	.49145	.37053	.34944
Q5FVG5	Tropomyosin 1	TPM2	.32989	.28146	.45933	.43703	.40284
Q6MFZ1	RT1 class I	RT1‐M1‐5	.33476	.31396	.39337	.38604	.40935
Q9P290	Solute carrier family 22 member 17	Slc22a17	.34682	.43023	.32001	.52572	.37683
Q8VIN2	Annexin	Q8VIN2	.35220	.64864	.64505	.34932	.42196
A0A0G2JWK7	Transgelin	Tagln	.36476	.40902	.35256	.37007	.60992
B2RZD4	60S ribosomal protein L34	Rpl34	.37585	.36258	.45139	.55468	.41238
A0A0G2K2V6	Keratin, type I cytoskeletal 10	Krt10	.38868	.47179	.18262	.63517	.34946
A0A0G2K6J5	Myosin light polypeptide 6	Myl6	.43160	.50325	.66767	.28007	.50899
Q9Z1P2	Alpha‐actinin‐1	Actn1	.45562	.56864	.47392	.45700	.49820
G3V6P7	Myosin, heavy polypeptide 9	Myh9	.47272	.33012	.55756	.53841	.39933
P68035	Actin, alpha cardiac muscle 1	Actc1	.47725	.51444	.54125	.37805	.48617
P02680	Fibrinogen gamma chain	Fgg	.56785	.39359	.25029	.33854	.24229
Q641Y0	Dolichyl‐diphosphooligosaccharide–protein glycosyltransferase 48 kDa subunit	Ddost	.64635	.42148	.29379	.39559	.43517
Up‐regulation							
G3V6G4	Recoverin	Rcvrn	1.52475	3.42520	1.90749	2.30165	3.81403
D3ZKX8	Family with sequence similarity 169, member A	Fam169a	1.55139	3.17679	2.77458	2.30530	3.00438
E2RUH2	Ribonuclease inhibitor	Rnh1	1.57229	2.16528	2.63320	1.52890	2.12205
P21575	Dynamin‐1	Dnm1	1.58527	2.11399	2.52607	1.92608	2.15384
Q9QYU4	Ketimine reductase mu‐crystallin	Crym	1.59123	1.53463	3.49471	2.48791	2.16249
D3ZLZ7	Inosine‐5′‐monophosphate dehydrogenase 1	Impdh1	1.61002	2.52125	1.61227	1.82501	3.23548
F1M471	EPM2A‐interacting protein 1	Epm2aip1	1.62951	1.73340	1.71811	1.69181	1.87136
Q5FVM4	Non‐POU domain‐containing octamer‐binding protein	Nono	1.64205	2.29589	2.47585	2.0761	2.34220
Q5XIE0	Acidic leucine‐rich nuclear phosphoprotein 32 family member E	Anp32e	1.64668	2.20588	1.88964	2.28429	2.50702
M0R3N4	Vesicle amine transport 1‐like	Vat1l	1.69109	2.09379	3.01540	2.17249	2.13362
A0A0G2JUX5	Transcriptional activator protein Pur‐beta	Purb	1.70230	2.80761	2.91012	2.05025	2.54624
O88767	Protein/nucleic acid deglycase DJ‐1	Park7	1.72324	2.70420	3.53109	2.25288	3.49859
O35796	Complement component 1 Q subcomponent‐binding protein	C1qbp	2.08203	1.84227	2.25983	1.74563	2.14898
P47819	Glial fibrillary acidic protein	Gfap	1.73027	1.98764	6.60683	4.69927	4.92120
P15887	S‐arrestin	Sag	1.8691	2.35355	2.73898	5.41263	6.33378
P12368	cAMP‐dependent protein kinase type II‐alpha regulatory subunit	Prkar2a	1.89630	2.57446	2.79823	2.25377	3.20962
F1LNC8	Interphotoreceptor matrix proteoglycan 2	Impg2	1.92785	1.62596	3.58728	2.44987	3.02114
F1LMW7	Myristoylated alanine‐rich C‐kinase substrate	Marcks	2.06343	2.14228	3.07641	1.97257	2.62340
P04631	Protein S100‐B	S100b	2.10259	2.42321	2.98445	3.07049	2.69259
G3V6H9	Nucleosome assembly protein 1‐like 1	Nap1/1	2.20634	3.55405	5.34293	3.81639	3.59727
P07335	Creatine kinase B‐type	Ckb	2.49786	2.38444	3.26172	2.45298	3.2785
Q6AYR8	Secernin‐2	Scrn2	3.10959	3.87169	2.94265	2.87996	2.74484
D4A4W7	Mitochondria‐localized glutamic acid	Mgarp	3.91372	4.41917	3.87734	3.13725	5.50719
A0A1K0FUA6	Globin a2	LOC689064	4.92857	4.46899	2.17224	4.90831	3.48620
A0A0G2JSH5	Serum albumin	Alb	6.19058	4.10714	4.22091	5.40322	5.35121

Expression of glial fibrillary acidic protein (GFAP) and complement component 1q (C1q) binding protein (gC1qR) increased, while GFAP over‐expressed in activated Müller cells after ONT‐induced RGC denaturation. To furthermore validate the expression of GFAP and gC1qR sequentially upregulated proteins in the retina, we performed western blot (Wb) analysis on the retinas of Sprague Dawley (SD) rats with or without ONT induced RGC denaturation and confirmed the difference between (p ＜ 0.05, Figure 2A). GFAP expression in the retina increased with the time after ONT and reached a peak at 14 days. Our results indicate that GFAP may be a disease‐specific biomarker for activated Müller cells in the retina and GS a key enzyme in glutamate metabolism can be a biology‐specific biomarker. Results from double immunofluorescence staining demonstrated that postONT GFAP and GS were co–localized at the end–foot and nerve fiber of Müller cells (Figure 2B), confirming that Müller cells were activated after ONT, rather than GS (Figure 2B, 2C).

**FIGURE 2:**
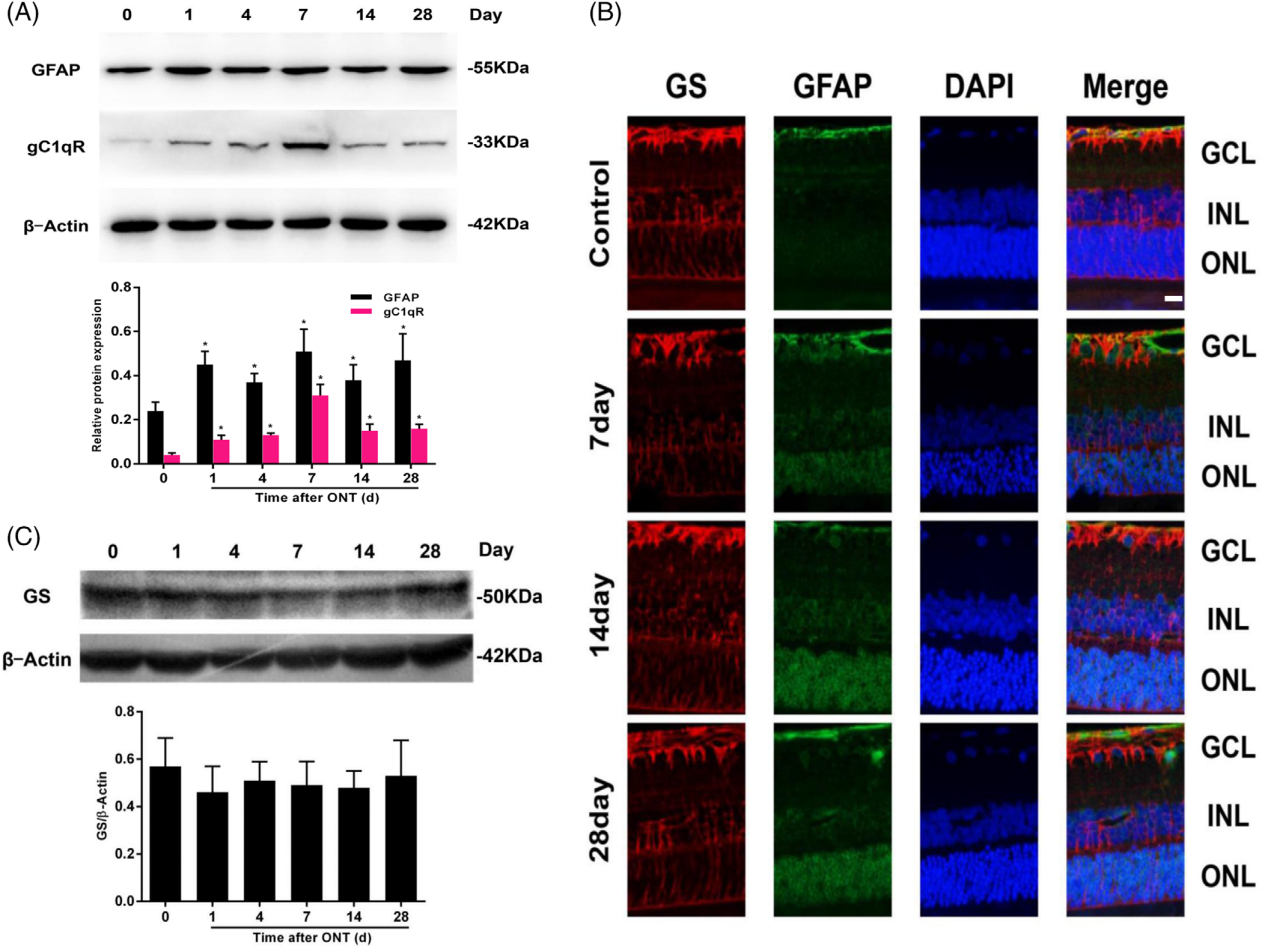
Expression and verification of GFAP, gC1qR and GS in the retina. (A) Glial fibrillary acidic protein (GFAP) and complement component 1q binding protein (gC1qR) in the retina after optic nerve transection at five time points were validated by western blot analysis and normalized to β‐actin for semi‐quantitative analysis. Data were represented into mean ± SD (*n* = 4). *p < .05, compared with control group. (B) Double immunofluorescence of glial fibrillary acidic protein (GFAP) and glutamine synthetase (GS) in the retina of rats after optic nerve transection (ONT). Retinal sections of the control group and the ONT group at days 7, 14, and 28 were stained with antibodies against GS (red) and GFAP (green). Nuclei were stained in blue using DAPI. (×400). (C) GS in the retina after optic nerve transection at five time points were analyzed by western blot and normalized to β‐actin for semi‐quantitative analysis. Data were represented into mean ± SD (*n* = 4). *p < .05, compared with control group. Scale bars = 25 μm. GCL: ganglion cell layer, INL: inner nuclear layer. ONL: outer nuclear layer

Glutamate metabolic pathways are altered after retinal RGC injury. L‐Glutamate/L‐aspartate transporter (GLAST) is a critical glutamate transporter to effectively remove excess glutamate from synaptic sites. We noticed that GLAST expression in rats without ONT was more widespread from the ganglion cell layer to the outer nuclear (ONL), especially in the outer plexiform layer. GLAST‐labelled Muller cell processes were diffused thoughout the ONL and GLAST expression increased from 7 days after ONT induction (Figure 3A). GLAST protein expression was significantly higher in rats with ONT group than those without ONT (Figure 3A, 3B).

**FIGURE 3:**
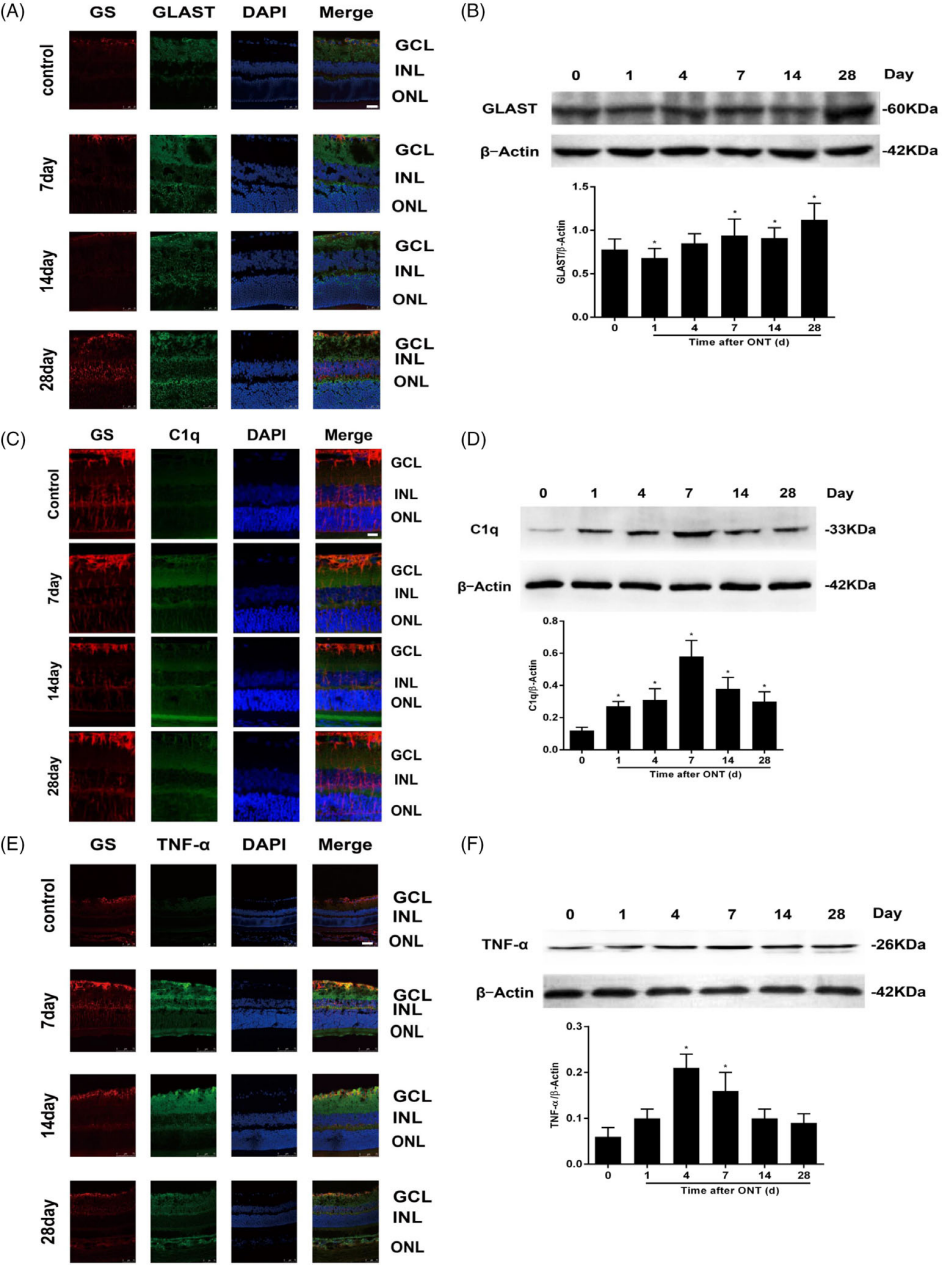
Expression and verification of GS, GLAST, C1q, and TNF‐α in retina. (A) Double immunofluorescence of glutamine synthetase (GS) and L‐glutamate/L‐aspartate transporter (GLAST) in the retina of rats after optic nerve transection (ONT). Retinal sections of the control group and the ONT group at days 7, 14, and 28 were stained with antibodies against GS (red) and GLAST (green). Nuclei were stained in blue using DAPI. (×400) (B) GLAST in the retina after optic nerve transection at five time points were analyzed by western blot and normalized to β‐actin for semi‐quantitative analysis. Data were represented into mean ± SD (*n* = 4). *p < .05, compared with control group. Scale bars = 25 μm. GCL: ganglion cell layer, INL: inner nuclear layer, ONL: outer nuclear layer. (C) Double immunofluorescence of glutamine synthetase (GS) and complement component 1q (C1q) in the retina of rats after optic nerve transection (ONT). Retinal sections of the control group and the ONT group at days 7, 14, and 28 were stained with antibodies against GS (red) and C1q (green). Nuclei were stained in blue using DAPI. (×400) (D) C1q in the retina after optic nerve transection at five time points were analyzed by western blot and normalized to β‐actin for semi‐quantitative analysis. Data were represented into mean ± SD (*n* = 4). *p < .05, compared with control group. Scale bars = 25 μm. GCL: ganglion cell layer, INL: inner nuclear layer, ONL: outer nuclear layer. (E) Double immunofluorescence of glutamine synthetase (GS) and tumour necrosis factor (TNF)‐α in the retina of rats after optic nerve transection (ONT). Retinal sections of the control group and the ONT group at days 7, 14, and 28 were stained with antibodies against GS (red) and TNF‐α (green). Nuclei were stained in blue using DAPI. (×400) (F) TNF‐α in the retina after optic nerve transection at five time points were analyzed by western blot and normalized to β‐actin for semi‐quantitative analysis. Data were represented into mean ± SD (*n* = 4). *p < .05, compared with control group. Scale bars = 25 μm. GCL: ganglion cell layer, INL: inner nuclear layer, ONL: outer nuclear layer

We furthermore evaluated the changes in C1q expression in the retina, which is the ligand of gC1qR and the first element of the classic complement activataion pathway. The interaction between C1q and gC1qR plays important roles in maintenance of the innate and acquired immunity and is closely associated with inflammation by initiating opsonization, amplifying recruiting phagocytes, and promoting membrance attack complex formation.^10^ In the normal retina, C1q is weakly expressed in RGCs and the inner and outer plexiform layers and obviously in Muller cell bodies and processes. We observed that C1q‐labelled Muller cell processes were diffuse throughout the INL and ONL, and C1q increased significantly after ONT induction (Figure 3C, 3D), as compared with Controls (p ＜ 0.05). We also found that tumor necrosis factor (TNF)‐α increased in the INL after ONT induction. TNF‐α mainly expressed in Muller cells and that TNF‐α protein expression was significantly higher by time after ONT than rats with ONT (p ＜ 0.05; Figure 3E, 3F).

In conclusion, Müller cells were activated following ONT‐induced RGC degeneration and accompanied by altered glutamate metabolism with the activation of classical complement pathways and inflammatory respones. Those reactions may contribute to RGC degeneration and provide new evidence to support the interaction between RGCs and Müller cells during primary and secondary RGC degeneration in the retina. Those alterations of key proteins can be a new class of targets for precision medicine therapy.

## CONFLICT OF INTEREST

The authors declare that there are no conflicts of interest.

## Supporting information

Figure S1
