# Supplementary figures and images for: Proteomic profiles of the retina in an experimental unilateral optic nerve transection: Roles of Müller cell activation
Source: Clin Transl Med. 2022 Apr 26;12(4):e631. doi: 10.1002/ctm2.631 (PMC9043120; doi:10.1002/ctm2.631)

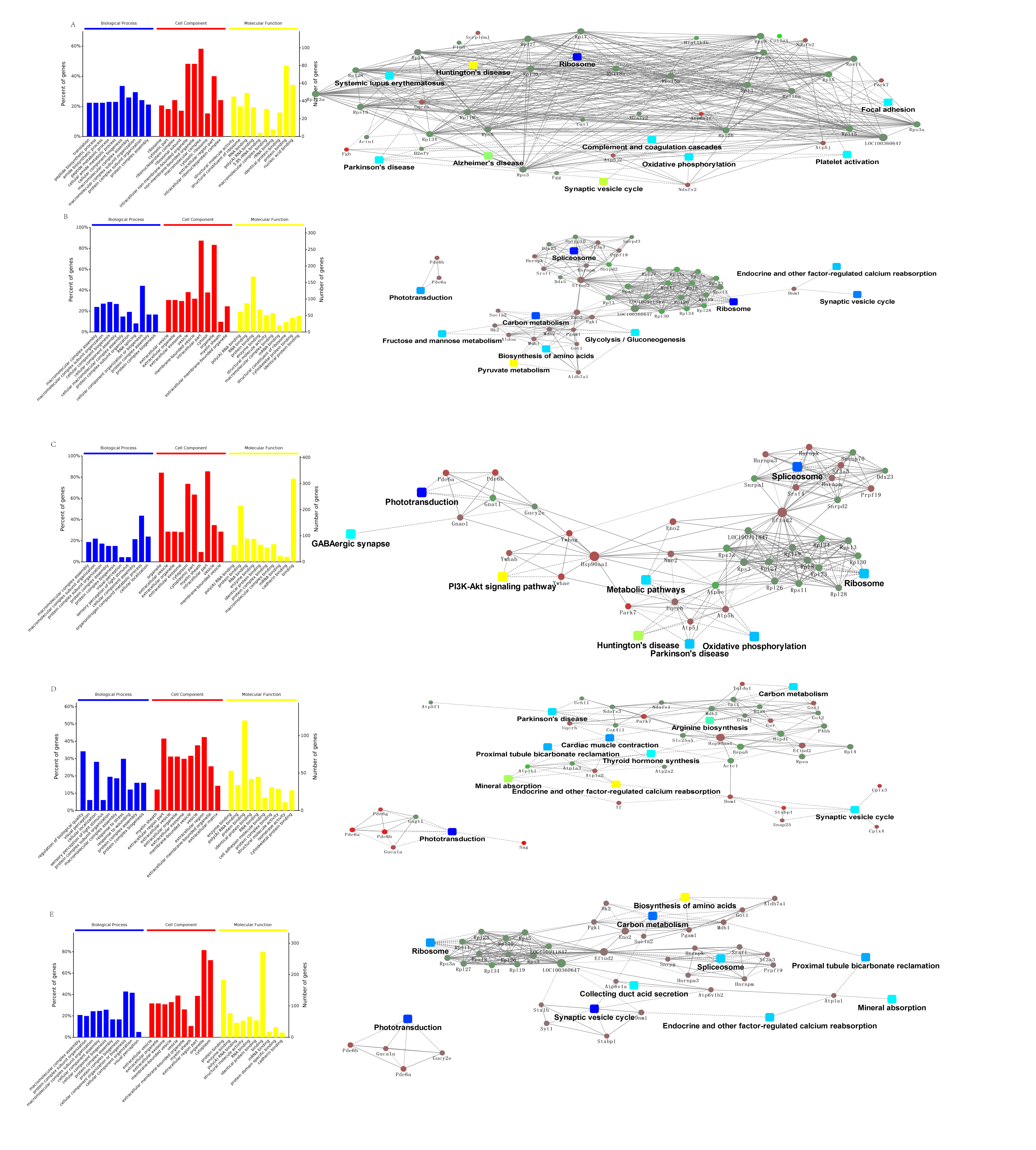

Supplement: Supplementary file 1 — Figure S1 [file CTM2-12-e631-s001.tif]
